# Cancer bioinformatics: A new approach to systems clinical medicine

**DOI:** 10.1186/1471-2105-13-71

**Published:** 2012-05-01

**Authors:** Duojiao Wu, Catherine M Rice, Xiangdong Wang

**Affiliations:** 1 Qingpu Branch, Fudan University Zhongshan Hospital, Shanghai, China; 2 Department of Pulmonary Medicine, Fudan University Zhongshan Hospital, Shanghai, China; 3 BMC Bioinformatics Executive Editor, BioMed Central, London, UK

## 

Cancer is one of the commonest causes of patient death in the clinic and a complex disease occurring in multiple organs per system, multiple systems per organ, or both, in the body. The poor diagnoses, therapies and prognoses of the disease could be mainly due to the variation of severities, durations, locations, sensitivity and resistance against drugs, cell differentiation and origin, and understanding of pathogenesis. With increasing evidence that the interaction and network between genes and proteins play an important role in investigation of cancer molecular mechanisms, it is necessary and important to introduce a new concept of Systems Clinical Medicine into cancer research, to integrate systems biology, clinical science, omics-based technology, bioinformatics and computational science to improve diagnosis, therapies and prognosis of diseases. Cancer bioinformatics is a critical and important part of the systems clinical medicine in cancer and the core tool and approach to carry out the investigations of cancer in systems clinical medicine. “Thematic Series on Cancer Bioinformatics” gather the strength of *BMC Bioinformatics*, *BMC Cancer*, *Genome Medicine* and *Journal of Clinical Bioinformatics* to headline the application of cancer bioinformatics for the development of bioinformatics methods, network biomarkers and precision medicine. The Series focuses on new developments in cancer bioinformatics and computational systems biology to explore the potential of clinical applications and improve the outcomes of patients with cancer.

## Expectations of methodologies

Cancer bioinformatics is one of multiple ways to concentrate bioinformatics methods in cancer, according to the specificity of disease metabolisms, signaling, communication, and proliferations. Clinical bioinformatics, an emerging science combining clinical informatics, bioinformatics, medical informatics, information technology, mathematics, and omics science together [1], can be considered to be one of critical elements addressing clinical relevant challenges in early diagnosis, efficient therapies, and predictive prognosis of patients with cancer. There is a need to develop cancer bioinformatics-specific methodologies or introduce new and advanced bioinformatics tools to answer the specific question of cancer. For example, the Semantic Web technology was used to understand high throughput clinical data and develop quantitative semantic models retrieved from Corvus, a data warehouse which provides a uniform interface to various forms of Omics data, based on systematic biological knowledge and by application of SPARQL endpoint [2]. Semantic models, containing genomic, transcriptomic and epigenomic data from melanoma samples with Gene Ontology data and regulatory networks constructed from transcription factor binding information, were applied for the interplay between a cell molecular state and its response to anti-cancer therapy. Multivariate assays, a process to characterize error introduced in the assay results from the intrinsic error in sample preparation and measurement of the contributing factors, were used to help and guide clinicians understanding the application to PAM50 centroid-based genomic predictors for breast cancer treatment plans and providing the uncertainty information in a usable way [3].

The applicability, specificity, and integration of methodologies, software, computational tools, and databases which can be used to explore the molecular mechanisms of cancer and identify and validate novel biomarkers, network biomarkers, and individualized medicine in cancer should be seriously considered. miRTrail is an integrative tool for analyzing comprehensive interactions of genes and miRNAs based on expression profiles to generate more robust and reliable results on deregulated pathogenic processes. It was suggested that miRTrail may open avenues for investigating the regulatory interactions between genes and miRNAs for human diseases, including cancer, by integrating information on 20.000 genes, almost 1.000 miRNAs, and roughly 280.000 putative interactions [4]. It would be helpful to explore the potential computational mode correlating such regulatory interactions between genes and miRNAs with clinical phenotypes, e.g. the variation of gene interactions among tumor locations, phages, differentiations, patient symptoms, or responses to therapies. Medical imaging should be one of important factors to be considered in the application of cancer bioinformatics, since the imaging in clinical pathology, ultrasonic, computerized tomography, nuclear magnetic resonance imaging, and positron emission tomography is one of the most necessary and important approaches in the “early and accurate” detection and diagnosis of cancer. Bioinformatic analyses of morphological features of masses and other abnormalities in medical images were initiated by selective extraction of target features by mathematical morphology and enhancement of the extracted features by two contrast modification techniques [5]. The algorithm described by Haustein and Schumacher in the **Thematic Series on Cancer Bioinformatics** in *Journal of Clinical Bioinformatics*[6] can simulate tumor growth and detect the formation of some metastases in advance of clinical detection in cells, on basis of clinical breast cancer data.

It may be a non-relative question or a future expectation how experts in cancer bioinformatics can help clinicians to establish the potential picture of gene or protein interactions and mechanisms correlated with tumor-associated shapes, densities, or locations. A Commentary by von der Heyde and Beissbarth in the **Thematic Series on Cancer Bioinformatics** in *BMC Medicine*[7] discusses the recent insights into mechanisms of cetuximab resistance in head and neck cancers resulting from novel analysis of the EGFR pathway.

## New strategies of biomarkers

Cancer bioinformatics is expected to play a more important role in the identification and validation of biomarkers, specific to clinical phenotypes related to early diagnoses, measurements to monitor the progress of the disease and the response to therapy, and predictors for the improvement of patient’s life quality. Of gene-, protein-, peptide-, chemical- or physic-based variables in cancer, biomarkers were investigated from a single one to multiple markers, from the expression to functional indication, and from the network to dynamic network. Network biomarkers as a new type of biomarkers with protein-protein interactions were investigated with the integration of knowledge on protein annotations, interaction, and signaling pathway. Alterations of network biomarkers can be monitored and evaluated at different stages and time points during the development of diseases, named dynamic network biomarkers, as one of the new strategies. Dynamic network biomarkers were expected to be correlated with clinical informatics, including patient complaints, history, therapies, clinical symptoms and signs, physician’s examinations, biochemical analyses, imaging profiles, pathologies and other measurements [8].

Systems clinical medicine is recommended as one of new strategies for the development of cancer biomarkers. Systems clinical medicine is coined as the integration of systems biology, clinical phenotypes, high-throughout technologies, bioinformatics and computational science to improve diagnosis, therapies and prognosis of diseases. Cancer biomarkers should possess the characters of networks, dynamics, interactions, and specificities to disease diagnosis, therapy and prognosis. Understanding the interaction between clinical informatics and bioinformatics is the first and critical step to discover and develop the new diagnostics and therapies for diseases. Such strategy has been described in other diseases like acute rejection after renal transplantation or lung diseases [9,10]. In brief, human samples from clinical studies under clear and strict criteria of participating recruitments are collected and harvested with an entire profile of clinical informatics translated from clinical descriptions. Gene and/or protein profiles of defined samples are analyzed and dynamic networks and interactions between genes and/or proteins can be figured out by bioinformatics and systems biology.

Selected disease-specific networks and dynamic networks of genes and/or proteins in patients are correlated with each of clinical phenotypes by the computational mode, to validate and optimize disease-special biomarkers. However, a number of challenges in the application of systems clinical medicine are encountered and need to be overcome; e.g. the optimal system to translate the information of clinical descriptions to clinical informatics, bioinformatics analysis oriented with disease severity, duration, location, sensitivity to therapies, and progress, or computational mode to integrate all elements from clinical and high-throughout data for precision conclusions. It is also a challenge to find out the variation and significance between molecular networks, between networks of molecules and clinical phenotypes, and between gene and/or protein interactions, in addition to the expression of genes and proteins. Cun and Fröhlich in the **Thematic Series on Cancer Bioinformatics** in *BMC Bioinformatics* report that incorporating protein network and interaction data improve the ability to interpret gene signatures in a study to stratify breast cancer patients, evidenced by findings that R weighted Recursive Feature Elimination and average pathway expression were most effective at generating interpretable signatures in those methods tested [11].

## Monitoring and prediction of precision medicine

Systems cancer medicine has been proposed as a new strategy towards realization of predictive, preventive, personalized and participatory (P4) medicine [12-15]. Tian et al. [15] recently proposed that a virtual cloud of billions of data generated from high-throughout technologies in patients would be figured out, including one or more disease-perturbed networks in cells of the relevant organ in the disease. Disease-perturbed molecular networks may indicate the abnormality of early signals and the functioning, to finally carry out P4 medicine in cancer. However, cancer clinical bioinformatics is an important way to reach systems clinical medicine by combining clinical measurements and signs with human cancer tissue-generated bioinformatics, understanding clinical symptoms and signs, disease development and progress, and therapeutic strategy, and mapping relationships that integrate discrete elements that collectively direct global function within a particular -omic category, with clinical examinations, pathology, biochemical analysis, imaging and therapies [1,8]. Ren and colleagues in the **Thematic Series on Cancer Bioinformatics** in *BMC Bioinformatics* have developed an algorithm named Optimization Tool for Clustering and Classification for multiple types of measurements, including proteomic and next generation sequencing data types [16]. Such method could successfully and effectively discover class of unknown cancer samples as class prediction in both breast cancer and leukemia data sets.

Cancer bioinformatics plays an important role in monitoring and predicting the efficiency and effectiveness of the precision medicine, which provides the safest and most effective therapeutic strategy based on the gene and protein variations of each subject. The semantic heterogeneity of the data generated from microarrays, proteomics, epigenetics and next generation sequencing, provided an ontology-based solution for querying distributed databases over service-oriented, model-driven infrastructures by integrating molecular, pathology, radiology and clinical data in an efficient manner [17]. A recent study performed a forward-genetic screen guided by genomic analysis of human hepatic cellular carcinoma, and found that a common genetic alteration in liver cancer (11q13.3 amplification) resulted in activation of FGF19 which caused the selective sensitivity to FGF19 inhibition through subsequent analysis with mouse models and RNAi [18]. It is expected to develop accurate tools for delivering the right treatment to the right patient in the right time, based on molecular network characters of each patient’s tumor. Cancer bioinformatics and systems biology are expected to improve prevention, diagnosis and treatment through therapy design. The classical techniques of statistics and bioinformatics for analysis of the genome, biological sequences, large-scale ‘omic’ data sets and protein three-dimensional structure could form an indispensable backbone for computational cancer research [19].

In conclusion, cancer bioinformatics as an emerging strategy is one of the most critical and useful approaches to systems clinical medicine for clinical research and applications and improve the outcomes of patients with cancer. The **Thematic Series on Cancer Bioinformatics** provides a unique and outstanding platform and opportunity for scientists to integrate omics science, bioinformatics tools and data, clinical research, disease-specific biomarkers, dynamic networks, with precision medicine, together fighting cancer and improving the life quality of patients with cancer.
